# An interpretable nomogram with SHAP analysis predicts thrombotic failure of forearm arteriovenous fistulas

**DOI:** 10.3389/fsurg.2026.1743314

**Published:** 2026-02-26

**Authors:** Yilin Xu, Linsen Jiang, Haixia Zhang, Rong Ni, Peng Qian, Zhi Wang, Weiwei Li

**Affiliations:** Department of Nephrology, The Second Affiliated Hospital of Soochow University, Suzhou, China

**Keywords:** arteriovenous shunt, surgical, explainable artificial intelligence, nomograms, renal dialysis, thrombosis

## Abstract

**Objective:**

End-stage renal disease is an increasing global health problem. Arteriovenous fistula (AVF) thrombosis is a major cause of access failure in maintenance hemodialysis (MHD) patients. An interpretable nomogram, integrated with SHapley Additive exPlanations (SHAP) analysis is developed and validated for predicting thrombotic failure of forearm AVFs in MHD patients.

**Methods:**

A single-center retrospective cohort study enrolled 302 MHD patients with dysfunctional forearm AVFs undergoing percutaneous transluminal angioplasty. Patients were randomly allocated to training (70%) and validation (30%) sets. Univariable and multivariable logistic regression identified independent predictors for AVF thrombosis. A nomogram was constructed and its performance evaluated by the area under the receiver operating characteristic curve, calibration, and decision curve analysis. SHAP analysis was applied to quantify feature importance and directionality in the validation set.

**Results:**

The final model identified hypertension history, frequent intradialytic hypotension, body mass index, total cholesterol, C-reactive protein, and intact parathyroid hormone as independent predictors. The nomogram demonstrated good discrimination, with AUCs of 0.80 (95% CI: 0.73–0.86) in the training set and 0.71 (95% CI: 0.59–0.83) in the validation set, along with satisfactory calibration and clinical utility. SHAP analysis revealed red cell distribution width-standard deviation as the most influential predictor for individual risk, highlighting a distinction between statistical significance and predictive contribution.

**Conclusion:**

This study presents an interpretable nomogram with robust performance for predicting AVF thrombosis. The integration of SHAP analysis enhances model transparency and clinical trust, providing a valuable tool for personalized risk assessment and potential targeting of preventive strategies in MHD patients. Further external validation is warranted.

## Introduction

End-stage renal disease (ESRD) is an increasing global health problem (1–3). Kidney replacement therapy including kidney transplantation and dialysis, such as hemodialysis and peritoneal dialysis can improve life quality of ESRD patients and offer a higher survival rate, making it an advantageous therapeutic option for the treatment of ESRD patients (4–6). Arteriovenous fistula (AVF) thrombosis is a major cause of access failure in maintenance hemodialysis (MHD) patients, frequently resulting in urgent interventions, permanent access loss, and increased healthcare utilization (7–9). While AVFs are the preferred vascular access owing to their superior long-term patency, their susceptibility to thrombosis underscores the need for proactive risk stratification (10–12).

Although numerous risk factors—spanning clinical, hemodynamic, and biomarker domains—have been associated with AVF thrombosis (13–15), their translation into clinically practical prediction tools remains inadequate. Existing models often demonstrate only moderate discriminatory power and suffer from limited external validation (16). Furthermore, many are derived from heterogeneous cohorts that combine distinct AVF types and failure etiologies (17), potentially compromising their generalizability to specific clinical scenarios. The “black-box” nature of some advanced algorithms further impedes clinical adoption by obscuring the reasoning behind predictions.

To address these limitations, we developed and validated a prediction model for thrombotic AVF failure specifically in MHD patients with forearm AVFs undergoing percutaneous transluminal angioplasty. By rigorously comparing patients with confirmed thrombosis against those with pure stenosis, we aimed to construct a practical tool using routinely available clinical data. A key methodological advancement is the incorporation of SHapley Additive exPlanations (SHAP) analysis, an explainable AI (XAI) framework. This integration not only facilitates risk prediction but also quantifies the contribution and directional effect of each predictor, thereby enhancing model transparency and clinical trust. This study provides an interpretable foundation for risk screening and highlights the utility of explainable modeling in developing robust, clinically integrated predictive solutions.

## Method

### Study design and participant

This single-center, retrospective cohort study enrolled consecutive MHD patients who underwent percutaneous transluminal angioplasty (PTA) for AVF dysfunction between July 15, 2020, and June 26, 2025. The decision to perform PTA followed the NKF-KDOQI Clinical Practice Guidelines for Vascular Access (2019 update) (18). Indications included: (1) clinical indicators of access dysfunction, such as prolonged post-puncture bleeding, difficult cannulation, or altered characteristics of the thrill or bruit; (2) hemodynamic criteria, defined as a >50% reduction in access blood flow (Qa) from baseline or an absolute Qa <500 mL/min (measured via ultrasound dilution or other validated techniques); and/or (3) duplex ultrasound evidence of ≥50% luminal stenosis at the arterial anastomosis, juxta-anastomotic segment, or outflow vein. Preoperative imaging was used to exclude central venous stenosis. The study was approved by the Institutional Review Board of the Second Affiliated Hospital of Soochow University, with waiver of informed consent due to its retrospective design. Inclusion criteria were: (1) age 18–80 years; (2) MHD duration ≥3 months; (3) dialysis frequency of three times weekly; and (4) presence of a forearm AVF. Exclusion criteria included: (1) MHD duration < 3 months; (2) non-thrice-weekly dialysis; (3) upper-arm AVF or arteriovenous graft; (4) AVF maturation time < 3 months; (5) prior AVF thrombosis; (6) severe infection within one month before PTA; or (7) incomplete clinical data. After screening, 302 eligible patients were included and randomly allocated to a training set (70%, *n* = 211) or a validation set (30%, *n* = 91) using a computer-generated random number sequence with a fixed seed to ensure reproducibility.

### Data collection

Data were retrospectively collected from the hospital's electronic medical records, laboratory information system, and hemodialysis management system. To ensure accuracy, two investigators independently extracted the data, with any discrepancies resolved through consensus. The collected variables included: (1) Sociodemographic characteristics: sex, age, blood pressure, and body mass index (BMI). (2) Comorbidities: history of diabetes, hypertension, coronary artery disease, heart failure, peripheral vascular disease, and cerebrovascular disease. (3) Laboratory parameters: All measurements were obtained from pre-dialysis venous blood samples drawn after the long interdialytic interval (typically 2–3 days) closest to the PTA procedure. Parameters included: Renal function and nutrition: serum creatinine (SCr), blood urea nitrogen (BUN), uric acid (UA), albumin (Alb); Anemia and iron metabolism: hemoglobin (Hb), red blood cell count (RBC), red cell distribution width (RDW); Coagulation profile: prothrombin time (PT), activated partial thromboplastin time (APTT), D-dimer; Dialysis adequacy: single-pool Kt/V (spKt/V).; Mineral and bone metabolism: intact parathyroid hormone (iPTH), corrected calcium (cCa), phosphate (P); Lipid profile: total cholesterol (TC), triglycerides (TG). (4) Dialysis and vascular access parameters: dialysis frequency, intradialytic hypotension (IDH) occurrence within one month before PTA (defined as episodes in ≥30% of sessions) (19, 20), and arterial/venous internal diameters (≥ 2.0 mm) measured by preoperative ultrasonography before initial AVF creation. IDH was defined per Kidney Disease Outcomes Quality Initiative guidelines as a systolic blood pressure decrease ≥20 mmHg or mean arterial pressure decrease ≥10 mmHg during hemodialysis, accompanied by clinical symptoms requiring intervention (21).

### Diagnostic criteria for autologous AVF thrombosis

Autologous AVF thrombosis was diagnosed according to established criteria (22), which required the presence of the following findings: (1) loss of the characteristic fistular thrill or pulsation; (2) absence of an audible bruit combined with induration of the fistula vein rendering it non-compressible; (3) potential signs of localized inflammation, such as swelling and pain; and (4) color Doppler ultrasonographic evidence of low- to medium-level echogenic material within the AVF lumen with an absence of blood flow signals.

### Statistical analysis

All statistical analyses were conducted using R software (version 4.3.0). The dataset was randomly partitioned into a training set (70%) and a validation set (30%) (23). Predictors were identified and a predictive model was developed through multivariable logistic regression in the training set, with subsequent performance evaluation conducted in the validation set. Continuous variables following normal distribution are presented as mean ± standard deviation and compared using independent samples *t*-test. Non-normally distributed variables are expressed as median with interquartile range and compared using Mann–Whitney *U*-test. Categorical variables are summarized as frequencies (percentages) and compared using Chi-square or Fisher's exact test as appropriate. Ordinal data were analyzed using Wilcoxon rank-sum test. Variables demonstrating association with AVF thrombosis at *P* < 0.20 in univariable analyses were entered into the multivariable model. A bidirectional stepwise selection approach, with the minimization of the Akaike Information Criterion (AIC) as the objective, was employed to identify independent predictors and construct the final model. All retained variables exhibited variance inflation factors (VIF) <2.0, indicating absence of substantial multicollinearity. The final model was presented as a nomogram. Model discrimination was quantified by the area under the receiver operating characteristic curve (AUC). Calibration was assessed via calibration plots and the Hosmer-Lemeshow test. Clinical utility was evaluated through decision curve analysis (DCA). To enhance interpretability and validate feature importance, SHapley Additive exPlanations (SHAP) values were computed on the independent validation set and interpreted using the shapviz package (version 0.10.2) in R (version 4.3.1). Statistical significance was defined as two-tailed *P* < 0.05.

### Ethical considerations

This study was approved by the Ethics Committee of the Second Affiliated Hospital of Soochow University (Approval No. LKzO2nO3B). The requirement for informed consent was waived due to the retrospective nature of the study.

## Results

### Characteristics of the participants

During the study period, a total of 508 patients underwent endovascular interventions for vascular access dysfunction at our center. The distribution of the intervened access types was as follows: forearm AVF in 383 cases (75.4%), upper-arm AVF in 110 cases (21.7%), and arteriovenous graft (AVG) in 20 cases (3.9%). This study strictly focused on the cohort of 302 patients with dysfunctional forearm AVFs who met the predefined inclusion and exclusion criteria (see the Methods section). These 302 maintenance hemodialysis patients were included and randomly assigned to training (*n* = 211) and validation (*n* = 91) sets in a 7:3 ratio. The cohort consisted of 201 males (66.6%) and 101 females (33.4%), with a median age of 65 years (interquartile range: 55–73). Thrombosis was documented in 98 patients (32.5%). Baseline characteristics showed no statistically significant differences between the training and validation sets (all *P* > 0.05) (Table 1).

**Table 1:** Baseline characteristics of the patients in the training and validation sets.

Variables	Total (*n* = 302)	Validation set (*n* = 91)	Training set (*n* = 211)	*Z/χ^2^/ t*	*P*
Age (year)	65.00 (55.00, 73.00)	64.00 (52.50, 72.00)	66.00 (56.00, 73.00)	1.30	0.194
Male, *n* (%)	201 (66.56)	57 (62.64)	144 (68.25)	0.90	0.343
BMI (kg/m^2^)	22.04 (19.21, 24.38)	22.41 (19.47, 24.36)	21.87 (18.85, 24.36)	−1.24	0.214
Dialysis vintage(month)	56.00 (26.00, 91.00)	46.00 (27.00, 78.00)	60.00 (26.00, 97.50)	−1.55	0.121
SBP (mmHg)	143.12 ± 27.12	140.47 ± 25.76	144.26 ± 27.67	−1.11	0.266
DBP (mmHg)	81.00 (73.00, 90.00)	80.00 (71.50, 90.50)	82.00 (74.00, 90.00)	−1.14	0.255
IDH≥30%, *n* (%)	39 (12.91)	14 (15.38)	25 (11.85)	0.71	0.400
Venous diameter≥2 mm, *n* (%)	141 (46.69)	40 (43.96)	101 (47.87)	0.39	0.532
Arterial diameter≥2 mm, *n* (%)	202 (66.89)	61 (67.03)	141 (66.82)	0.00	0.972
spKt/V	1.40 ± 0.13	1.41 ± 0.14	1.40 ± 0.12	0.61	0.545
Serum creatinine (umol/L)	867.28 ± 236.79	861.10 ± 227.31	869.95 ± 241.25	−0.30	0.766
Albumin (g/L)	39.09 ± 4.92	38.39 ± 4.98	39.38 ± 4.87	−1.62	0.107
Hemoglobin (g/L)	112.85 ± 20.39	109.53 ± 21.88	114.29 ± 19.59	−1.87	0.062
Uric acid (umol/L)	377.50 (315.25, 464.00)	395.00 (338.00, 466.50)	371.00 (304.50, 461.00)	−1.24	0.215
Blood urea nitrogen (mmol/L)	21.15 (17.70, 26.75)	21.60 (17.90, 27.00)	20.70 (17.70, 26.45)	−0.39	0.697
iPTH (pg/mL)	236.97 (139.07, 350.28)	265.04 (143.14, 391.11)	226.77 (138.26, 336.80)	−1.03	0.304
Corrected calcium (mmol/L)	2.21 (2.09, 2.33)	2.21 (2.08, 2.33)	2.21 (2.10, 2.33)	−0.19	0.850
Phosphate (mmol/L)	1.83 (1.49, 2.28)	1.84 (1.48, 2.35)	1.82 (1.50, 2.22)	−0.33	0.741
Total cholesterol (mmol/L)	3.64 (2.90, 4.33)	3.63 (2.96, 4.21)	3.67 (2.84, 4.42)	−0.55	0.585
Triglycerides (mmol/L)	1.40 (0.99, 2.10)	1.51 (1.00, 2.09)	1.35 (0.98, 2.09)	−1.07	0.283
C-reactive protein (mg/L)	5.50 (5.20, 7.50)	5.60 (5.20, 7.80)	5.50 (5.20, 7.50)	−0.54	0.586
RDW-CV (%)	14.40 (13.60, 15.70)	14.40 (13.70, 16.05)	14.40 (13.55, 15.60)	−0.33	0.741
RDW-SD (fl)	49.10 (45.92, 52.38)	49.10 (46.50, 52.45)	49.10 (45.60, 52.35)	−0.50	0.619
PT (s)	13.25 (12.70, 13.90)	13.30 (12.75, 13.90)	13.20 (12.60, 13.90)	−0.55	0.583
APTT (s)	36.30 (31.20, 40.30)	36.20 (31.65, 40.55)	36.40 (31.15, 39.90)	−0.14	0.886
D-dimer (mg/L)	0.72 (0.40, 1.19)	0.78 (0.39, 1.23)	0.68 (0.41, 1.16)	−0.50	0.620
Cardiovascular disease, *n* (%)	81 (26.82)	20 (21.98)	61 (28.91)	1.56	0.212
Cerebrovascular disease, *n* (%)	51 (16.89)	14 (15.38)	37 (17.54)	0.21	0.647
Hypertension, *n* (%)	270 (89.40)	81 (89.01)	189 (89.57)	0.02	0.884
Diabetes, *n* (%)	123 (40.73)	44 (48.35)	79 (37.44)	3.14	0.077

Data are presented as mean ± standard deviation, median (interquartile range), or number (percentage). Between-group comparisons were performed using the independent samples *t*-test, Mann–Whitney *U*-test, χ^2^ test, or Fisher's exact test, as appropriate. BMI, body mass index; SBP, systolic blood pressure; DBP, diastolic blood pressure; IDH≥30%, intradialytic hypotension frequency ≥30% of dialysis sessions; spKt/V, single-pool Kt/V; iPTH, intact parathyroid hormone; RDW-CV, red cell distribution width coefficient of variation; RDW-SD, red cell distribution width standard deviation; PT, prothrombin time; APTT, activated partial thromboplastin time.

### Endovascular procedures and stenosis characteristics

All endovascular interventions in this cohort consisted of balloon PTA only; no stent placement was performed. The stenosis locations were: anastomotic (*n* = 94, 31.1%), juxta-anastomotic (*n* = 132, 43.7%), and peripheral venous (*n* = 76, 25.2%). Patients with central venous stenosis were identified during preoperative duplex ultrasound evaluation and were not included in this cohort, consistent with our focus on forearm AVF-specific lesions.

### Independent predictors of arteriovenous fistula thrombosis

Univariable analysis revealed significant associations between AVF thrombosis and multiple factors (*P* < 0.05), including BMI, uric acid, serum creatinine, blood urea nitrogen, total cholesterol, triglycerides, C-reactive protein, and intradialytic hypotension frequency. Variables showing association at *P* < 0.20 were included in multivariable analysis to minimize omission of potential predictors. After confirming absence of multicollinearity (all *VIF*s < 2.0), bidirectional stepwise selection identified a final model containing hypertension history, frequent intradialytic hypotension, BMI, uric acid, serum creatinine, parathyroid hormone, total cholesterol, C-reactive protein, and red cell distribution width-standard deviation (RDW-SD). The final analysis established hypertension history (*OR* = 9.66), frequent intradialytic hypotension (*OR* = 0.22), BMI (*OR* = 0.86), total cholesterol (*OR* = 1.47), C-reactive protein (*OR* = 1.02), and parathyroid hormone (*OR* = 1.01) as independent predictors for AVF thrombosis (Tables 2, 3).

**Table 2:** Univariate analyses of risk factors for arteriovenous fistula thrombosis in hemodialysis patients in the training set (*n* = 211).

Variables (Per unit increase)	*β*	OR (95%*CI*)	*P*
Age (year)	−0.01	0.99 (0.97–1.01)	0.344
Male, *n* (%)	−0.14	0.87 (0.47–1.61)	0.656
BMI (kg/m^2^)	−0.09	0.91 (0.84–0.99)	0.021
Dialysis vintage (month)	0.00	1.00 (1.00–1.01)	0.236
SBP (mmHg)	0.00	1.00 (0.99–1.02)	0.381
DBP (mmHg)	0.01	1.01 (0.99–1.03)	0.352
IDH≥30%, n(%)	1.33	3.76 (1.59–8.91)	0.003
Venous diameter≥2 mm, *n* (%)	0.21	1.24 (0.69–2.21)	0.470
Arterial diameter≥2 mm, *n* (%)	−0.33	0.72 (0.39–1.31)	0.283
spKt/V	−0.25	0.78 (0.07–8.18)	0.837
Serum creatinine (umol/L)	0.01	1.01 (1.01–1.01)	0.008
Albumin (g/L)	−0.02	0.98 (0.92–1.04)	0.520
Hemoglobin (g/L)	0.00	1.00 (0.99–1.02)	0.704
Uric acid (umol/L)	0.01	1.01 (1.01–1.01)	0.003
Blood urea nitrogen (mmol/L)	0.05	1.05 (1.01–1.09)	0.023
iPTH (pg/mL)	0.00	1.00 (1.00–1.00)	0.186
Corrected calcium (mmol/L)	0.10	1.10 (0.27–4.52)	0.895
Phosphate (mmol/L)	0.32	1.38 (0.87–2.18)	0.174
Total cholesterol (mmol/L)	0.28	1.32 (1.01–1.74)	0.046
Triglycerides (mmol/L)	0.35	1.43 (1.08–1.88)	0.013
C-reactive protein (mg/L)	0.02	1.02 (1.01–1.03)	0.014
RDW-CV (%)	0.10	1.10 (0.95–1.29)	0.207
RDW-SD (fl)	0.03	1.03 (0.98–1.08)	0.184
PT(s)	0.03	1.03 (0.98–1.08)	0.314
APTT (s)	−0.03	0.97 (0.93–1.00)	0.083
D-dimer (mg/L)	0.09	1.09 (0.95–1.25)	0.228
Cardiovascular disease, *n* (%)	−0.40	0.67 (0.35–1.30)	0.236
Cerebrovascular disease, *n* (%)	−0.14	0.87 (0.40–1.88)	0.720
Hypertension, *n* (%)	1.20	3.32 (0.95–11.64)	0.061
Diabetes, *n* (%)	−0.33	0.72 (0.39–1.33)	0.293

APTT, activated partial thromboplastin time; BMI, body mass index; DBP, diastolic blood pressure; IDH≥30%, frequent intradialytic hypotension (occurring in ≥30% of dialysis sessions); iPTH, intact parathyroid hormone; PT, prothrombin time; RDW-CV, red cell distribution width coefficient of variation; RDW-SD, red cell distribution width standard deviation; SBP, systolic blood pressure; spKt/V, single-pool Kt/V.

**Table 3:** Multivariate analyses of risk factors for arteriovenous fistula thrombosis in hemodialysis patients in the training set.

Variables (per unit increase)	*β*	OR (95%*CI*)	*P*
Intercept	−7.18	0.00 (0.00–0.08)	0.002
BMI (kg/m^2^)	−0.15	0.86 (0.78–0.95)	0.002
IDH≥30%, *n* (%)	1.50	4.50 (1.60–12.65)	0.004
Serum creatinine (umol/L)	0.00	1.00 (1.00–1.00)	0.055
Uric acid (umol/L)	0.00	1.00 (1.00–1.01)	0.072
iPTH (pg/mL)	0.01	1.01 (1.01–1.01)	0.040
Total cholesterol (mmol/L)	0.38	1.47 (1.01–2.13)	0.042
C-reactive protein (mg/L)	0.02	1.02 (1.01–1.04)	0.023
RDW-SD (fl)	0.05	1.05 (0.99–1.11)	0.094
Hypertension, *n* (%)	2.27	9.66 (1.86–50.18)	0.007

BMI, body mass index; SBP, systolic blood pressure; IDH≥30%, the frequency of intradialytic hypotension occurring in ≥30% of dialysis sessions; iPTH, intact parathyroid hormone; RDW-SD, red cell distribution width—standard deviation.

### Development and validation of a predictive nomogram

**Figure 1:**
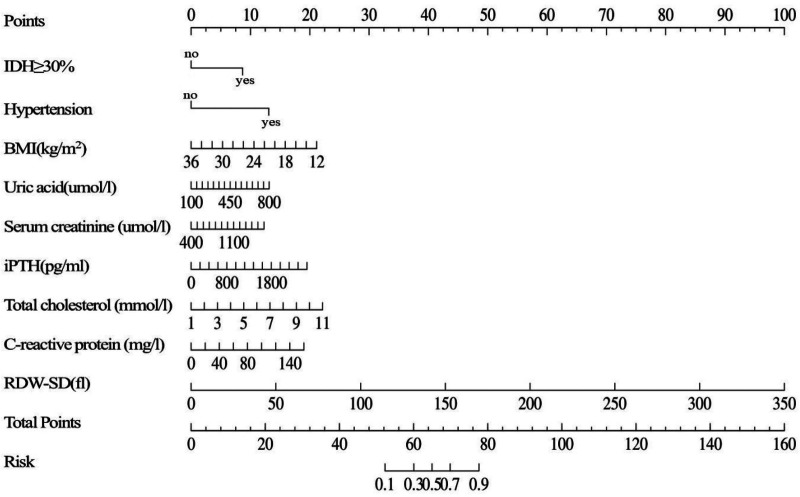
Nomogram for predicting thrombotic risk derived from the training set of maintenance hemodialysis patients (*n* = 211). IDH≥30%: the frequency of intradialytic hypotension occurring in ≥30% of dialysis sessions; BMI: body mass index; iPTH: intact parathyroid hormone; RDW-SD: red cell distribution width—standard deviation.

A multivariate logistic regression model was developed to predict the risk of thrombotic AVF failure and subsequently presented as a clinically applicable nomogram (Figure 1). The model incorporated the following predictors: history of hypertension, frequent IDH, BMI, UA, SCr, iPTH, TC, CRP, and RDW-SD.

**Figure 2:**
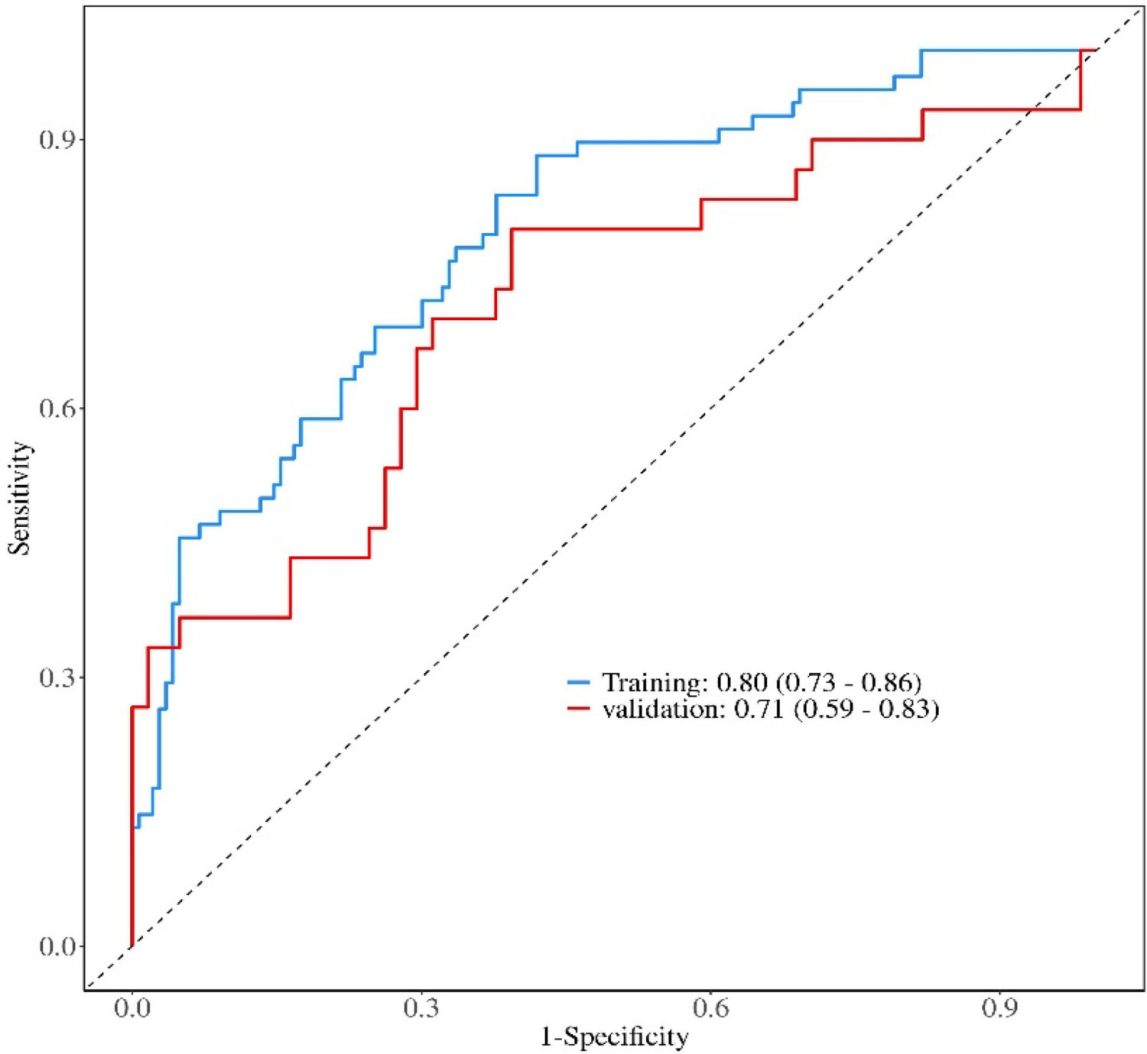
Receiver operating characteristic curves of the nomogram in the training and validation sets.

**Figure 3:**
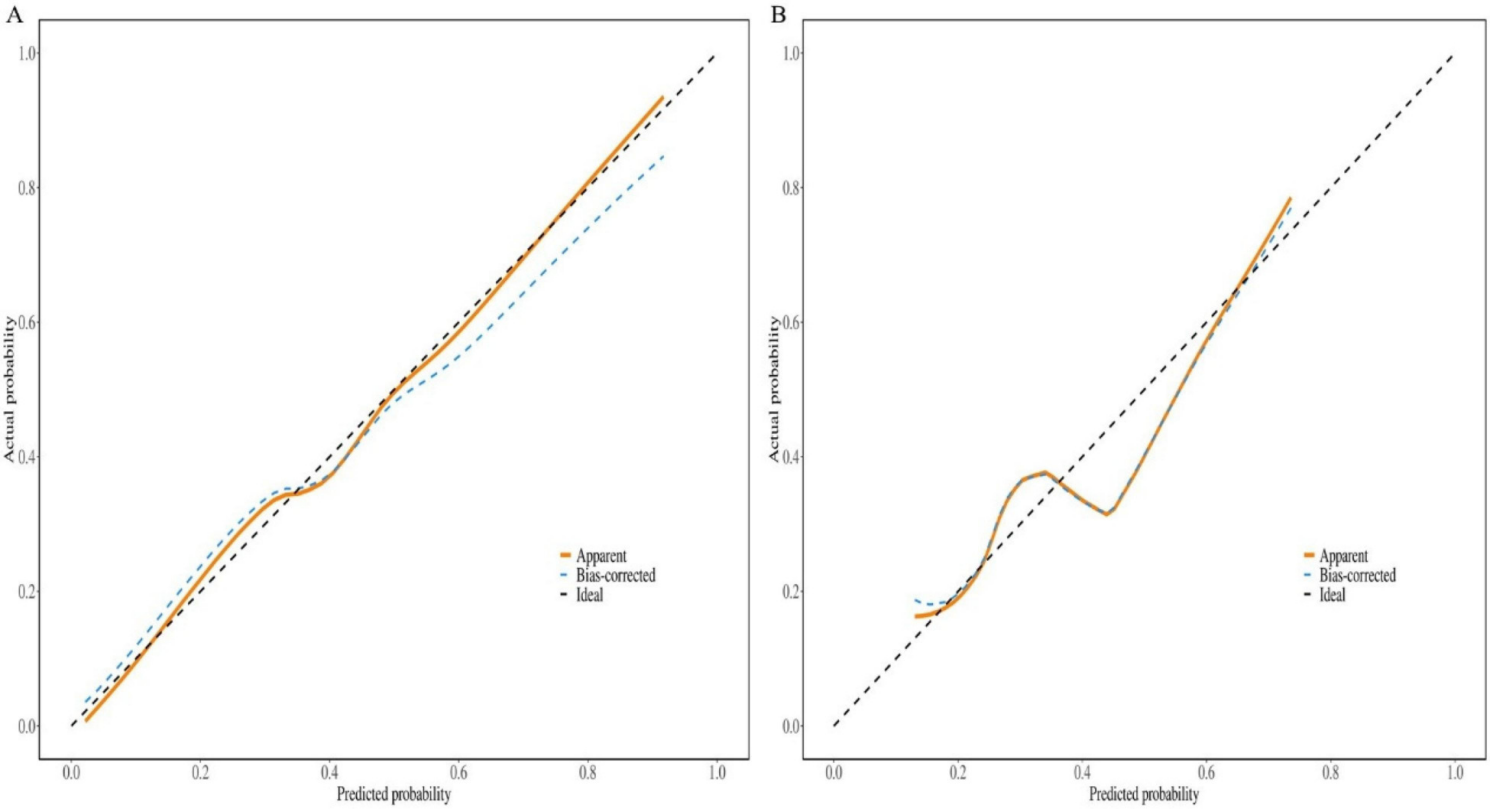
Calibration curve of the nomogram in the two sets. **(A)** Training set; **(B)** validation set.

The nomogram demonstrated good discriminative ability in the training set, with an *AUC* of 0.80 (95% *CI*: 0.73–0.86). Acceptable discrimination was maintained in the independent validation set, yielding an *AUC* of 0.71 (95% *CI*: 0.59–0.83) (Figure 2). Calibration curves showed satisfactory agreement between predicted probabilities and observed outcomes (Figure 3). The Hosmer-Lemeshow test indicated good model fit (training set: *P* = 0.25; validation set: *P* = 0.44).

At the optimal cutoff value of 0.211 determined by Youden's index, the model achieved a sensitivity of 0.88 (95% *CI*: 0.81–0.96) and a specificity of 0.57 (95% *CI*: 0.49–0.65) in the training cohort. Corresponding values in the validation cohort were 0.80 (95% *CI*: 0.66–0.94) for sensitivity and 0.49 (95% *CI*: 0.37–0.62) for specificity (Table 4).

**Table 4:** Performance metrics of the thrombotic AVF failure prediction nomogram in the training and validation sets.

Data	AUC (95%CI)	Accuracy (95%CI)	Sensitivity (95%CI)	Specificity (95%CI)	PPV (95%CI)	NPV (95%CI)	Cut off
Training	0.80 (0.73–0.86)	0.67 (0.61–0.74)	0.88 (0.81 −0.96)	0.57 (0.49–0.65)	0.50 (0.41–0.58)	0.91 (0.85–0.97)	0.211
Validation	0.71 (0.59–0.83)	0.59 (0.49–0.70)	0.80 (0.66 −0.94)	0.49 (0.37–0.62)	0.44 (0.31–0.57)	0.83 (0.71–0.96)	0.211

AUC, area under the receiver operating characteristic curve; PPV, positive predictive value; NPV, negative predictive value. Performance was evaluated at the optimal cutoff probability of 0.211 as determined by Youden's index.

This nomogram provides a practical risk assessment tool by enabling the summation of individual variable scores into a total points value, which is then converted into a thrombosis probability. Patients with a predicted probability exceeding 0.211 are classified as high-risk and may be considered for intensified monitoring or preventive interventions.

### Clinical practicality

**Figure 4:**
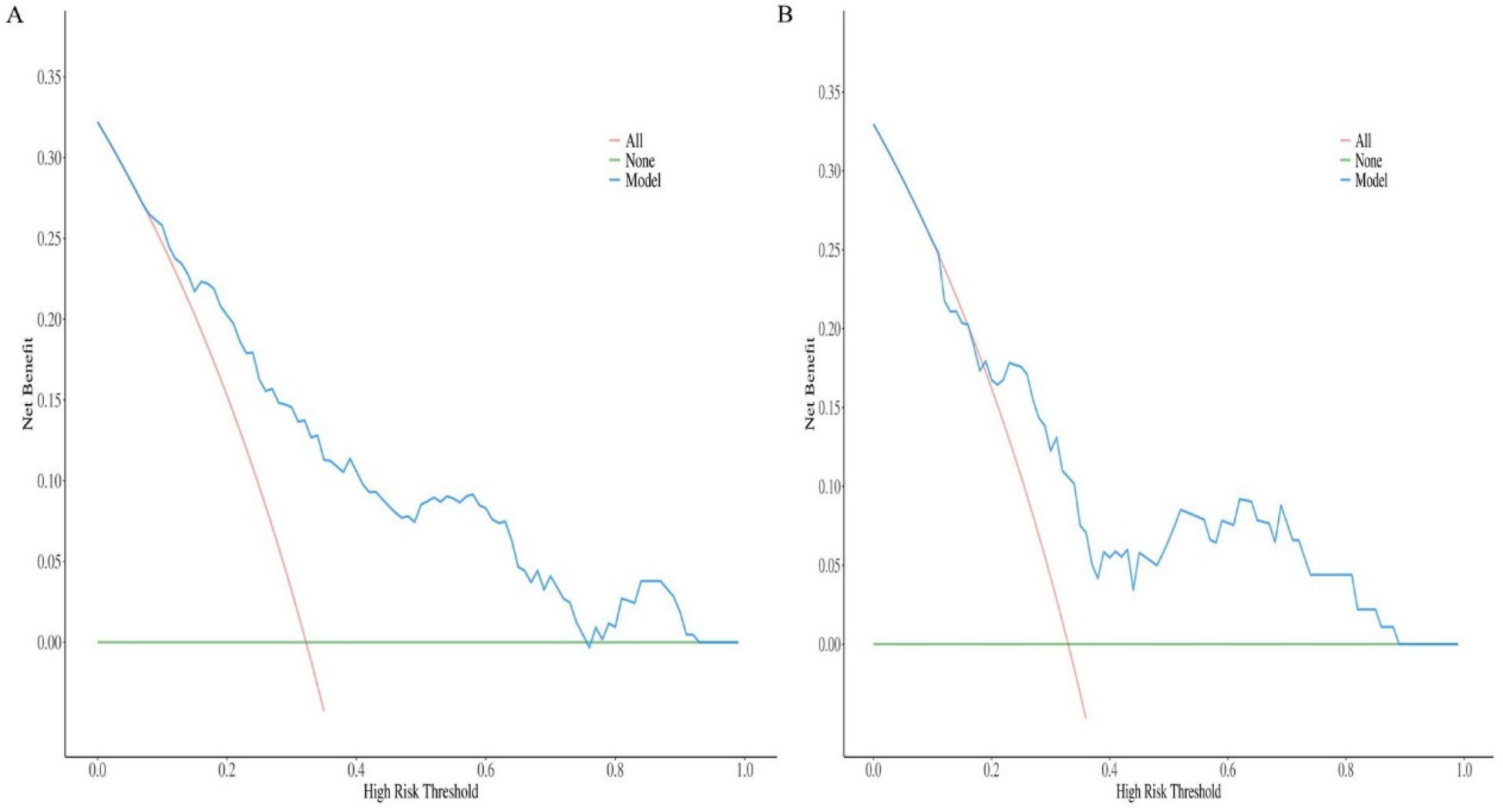
Decision curve analysis plot of the nomogram in the two sets. **(A)** Training set; **(B)** validation set.

DCA was performed to evaluate the clinical utility of the nomogram by quantifying the net benefit across a range of threshold probabilities. In both the training and validation sets, the DCA demonstrated that the prediction model provided a superior net benefit compared to the strategies of intervening on all patients or on no patients across the majority of clinically reasonable threshold probabilities (approximately 5%–100%) (Figure 4). This indicates that using the nomogram to guide clinical decisions on intervention for AVF thrombosis is potentially beneficial over a wide range of risk thresholds.

### Model interpretability and feature analysis based on SHAP

**Figure 5:**
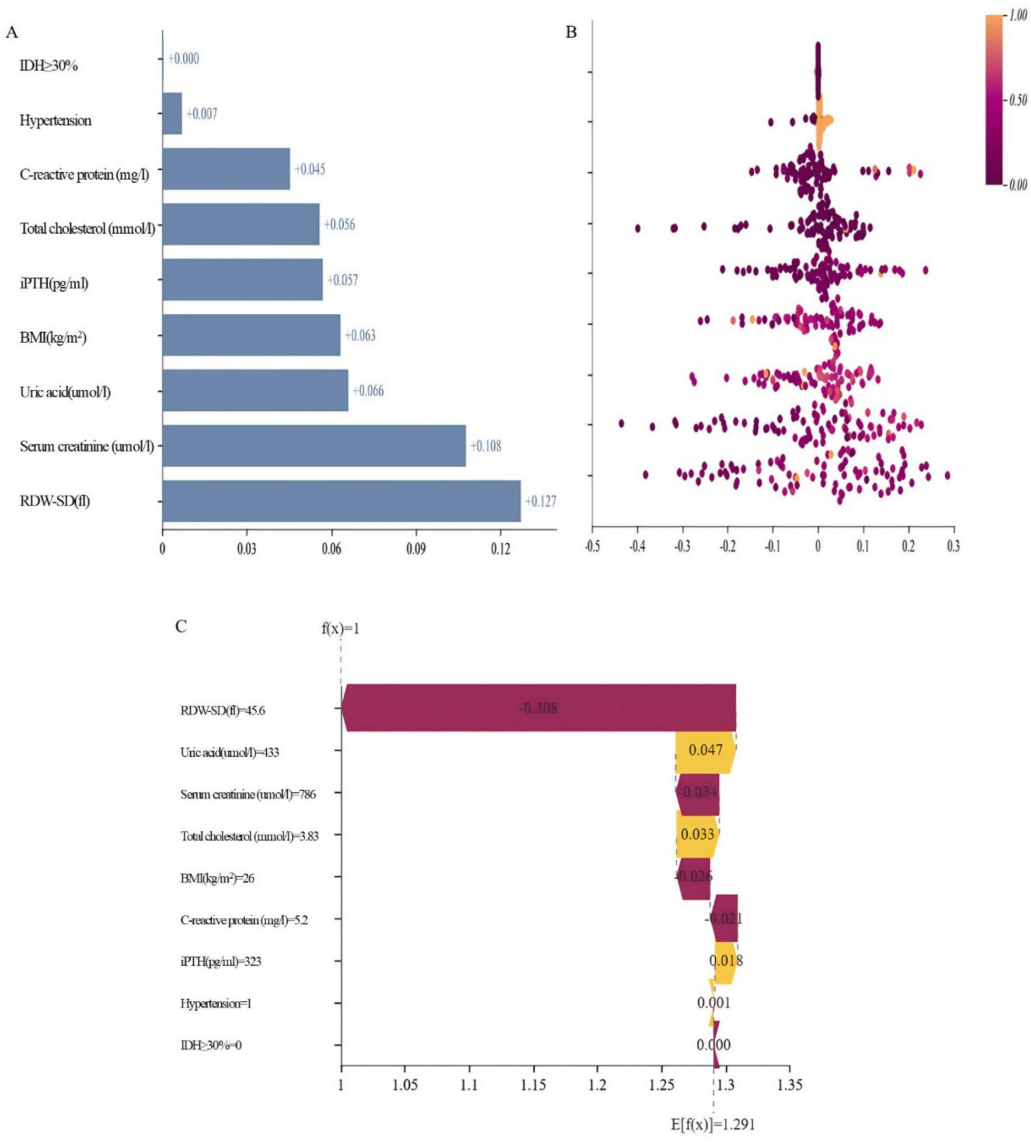
Model interpretability and feature analysis based on SHAP. **(A)** Feature importance was assessed using the mean absolute SHAP value. **(B)** The direction of feature effects was illustrated using a beeswarm plot. **(C)** SHAP force plot for a high-risk patient.

SHAP analysis on the independent validation set quantified variable contributions to the nomogram's predictions. Feature importance, assessed by mean absolute SHAP values (Figure 5A), identified RDW-SD (0.127), Scr (0.108), and UA (0.066) as the most influential predictors. Remaining variables showed progressively lower impacts: BMI (0.063), iPTH (0.057), TC (0.056), CRP (0.045), hypertension history (0.007), and IDH (0.000). The beeswarm plot (Figure 5B) illustrates effect directions, where elevated RDW-SD, creatinine, uric acid, TC, CRP, and iPTH values correlated with increased thrombosis risk (positive SHAP values). Conversely, hypertension history demonstrated protective associations (negative SHAP values), while IDH showed neutral impact.

## Discussion

Hemodialysis is the most usually utilized modality for renal replacement treatment worldwide (24–26). This study developed and validated an interpretable nomogram incorporating SHAP analysis for predicting thrombotic AVF failure in a rigorously defined cohort of MHD patients with forearm AVFs. The model demonstrated robust and consistent performance, with AUCs of 0.80 and 0.71 in the training and validation sets, respectively, satisfactory calibration, and a positive net benefit across a wide range of threshold probabilities, supporting its potential utility for clinical risk stratification.

Our findings regarding established risk factors—such as frequent IDH, elevated CRP, high TC, elevated iPTH, and low BMI—are consistent with existing pathophysiological understanding (27–29). IDH may promote thrombosis through recurrent fistula hypoperfusion (19), while CRP and TC reflect systemic inflammation, lipid metabolism disorder and dyslipidemia that contribute to a pro-thrombotic state (30–33). The associations of low BMI and high iPTH with thrombosis risk further underscore the influence of malnutrition-inflammation complex and mineral bone disease on vascular health in MHD patients (34).

The interpretation of hypertension history presented a compelling paradox: it was independently associated with increased thrombosis risk in the multivariable logistic regression model yet exhibited a protective association in the SHAP analysis. This divergence is not contradictory but rather exemplifies the complementary nature of traditional statistics and explainable AI. The regression model correctly identifies the net adverse etiological effect of hypertension at the population level. In contrast, SHAP explains individual predictions by capturing context-specific interactions. In our validation cohort, which bears a substantial burden of intradialytic hypotension, the higher baseline perfusion pressure conferred by a history of hypertension may become functionally protective, overriding its long-term atherogenic risk to maintain fistula patency (35). This immediate hemodynamic benefit—a manifestation of “reverse epidemiology” in end-stage renal disease—is precisely the nuanced, conditional reality that SHAP is designed to surface, and which may be masked in a population-average model. The potential confounding by unmeasured antihypertensive medication regimens (e.g., RAAS inhibitors) (36, 37) remains a key limitation. Therefore, integrating both methodologies provides a more complete picture: the regression model defines the underlying pathological risk, while SHAP elucidates the context-dependent clinical expression of that risk. Notably, SHAP analysis in this study suggested that the contribution of hypertension to AVF dysfunction risk varies across individuals, possibly reflecting underlying patient subgroup heterogeneity that is not fully appreciated. Exploratory analyses indicated that the effect of hypertension might differ depending on IDH status, although the limited sample size in the frequent-IDH subgroup precluded robust confirmation of this interaction, warranting validation in larger-scale studies.

The presented nomogram demonstrated high sensitivity (0.88 in training, 0.80 in validation) with moderate specificity (0.57 and 0.49, respectively). This performance profile was intentional: to minimize missed high-risk cases given the severe consequences of undetected AVF thrombosis. In practice, a negative result can reliably support routine monitoring. Conversely, a positive result should be interpreted as a high-sensitivity alert prompting intensified surveillance or evaluation of preventive measures, rather than a definitive diagnostic signal. This underscores the model's primary role as a screening and risk-stratification tool within a holistic clinical workflow.

A key methodological innovation of our study is the integration of SHAP-based explainable AI (XAI). SHAP analysis revealed a critical distinction between statistical significance and predictive contribution (38, 39). Notably, RDW-SD emerged as the most influential predictor in the validation set, despite not being statistically significant in the multivariable model. This finding highlights RDW-SD—a low-cost, routinely available marker of inflammation, oxidative stress, and nutritional status (37, 40, 41)—as a valuable component in thrombosis risk stratification. It also demonstrates how XAI can uncover clinically relevant predictors that might otherwise be overlooked based solely on conventional regression results. This aligns with the forefront of medical AI research, where studies increasingly emphasize that the high predictive contribution of a variable, as quantified by XAI, can be as clinically informative as its traditional statistical significance (42–44). Our work thus resonates with the evolving paradigm that values model interpretability and feature effect transparency for building clinical trust, a priority highlighted in recent state-of-the-art frameworks that combine ensemble models with SHAP explanations.

The combination of the nomogram and SHAP explanation enhances the model's translational potential (45, 46). This approach not only provides individualized risk estimation but also clarifies the reasoning behind each prediction, making it suitable for integration into clinical decision support systems (CDSS). For instance, in a dialysis center, the nomogram could be embedded within the electronic medical record (EMR) system. When a patient's routine data (e.g., BMI, CRP, RDW-SD) are entered, the system would automatically calculate a thrombosis risk score. For patients identified as high-risk, the accompanying SHAP analysis could visually display which factors most contributed to their risk, empowering clinicians to initiate personalized preventive measures, such as optimizing dry weight to reduce IDH frequency or intensifying anti-inflammatory management. This mirrors the application of AI in other high-stakes clinical decision scenarios, such as the use of AI-OCT systems in PCI surgery to automatically generate diagnostic reports and surgical strategy recommendations.

Several limitations should be considered. First, the single-center retrospective design and the moderate sample size may introduce selection bias and limit the stability of variable estimates. Although internal validation showed promising performance, large-scale, multicenter collaboration is essential to confirm generalizability across diverse populations and clinical settings. Second, the lack of detailed data on antihypertensive or anticoagulant medications, dynamic blood pressure trends, and AVF-specific characteristics (e.g., anastomotic configuration and flow parameters) may limit the model's comprehensiveness and precision (47). Third, the discrepancy between the regression and SHAP results for certain variables may reflect dataset shifts or more complex, non-linear relationships not fully captured by logistic regression.

Future research should focus on: (1) external validation through large-scale multicenter collaborations to enhance model generalizability and robustness, as exemplified by recent multi-center trials of AI systems in cardiovascular imaging; (2) refinement of the model with more granular data, including medication details and access characteristics; (3) exploration of non-linear machine learning approaches to better capture complex variable interactions, while maintaining interpretability via SHAP; (4) development of dynamic prediction models incorporating longitudinal data; and (5) prospective intervention studies to evaluate whether nomogram-guided management improves clinical outcomes. Furthermore, the interpretable framework developed here—combining a clinical prediction model with SHAP—holds significant promise for application beyond AVF thrombosis. It can be adapted to predict failure risks in other vascular access types, such as arteriovenous grafts (AVGs) and central venous catheters, which are also critical for hemodialysis and other long-term therapies. Establishing the utility of this XAI-driven approach across the spectrum of vascular access care could fundamentally improve personalized management strategies for all patients requiring durable vascular access.

## Conclusion

In summary, this study presents a clinically applicable and interpretable prediction model for AVF thrombosis that integrates traditional statistical methods with explainable AI. While acknowledging its limitations, we demonstrate the value of combining nomogram visualization with SHAP analysis to enhance both predictive performance and clinical interpretability. The findings underscore the potential of this approach to support personalized risk assessment and inform targeted intervention strategies in MHD patients, pending further validation and refinement in broader clinical settings.

## Data Availability

The raw data supporting the conclusions of this article will be made available by the authors, without undue reservation.
